# Forty-year hydropower generation reanalysis for Conterminous United States

**DOI:** 10.1038/s41597-025-05323-y

**Published:** 2025-06-19

**Authors:** Sean W. D. Turner, Debjani Singh, Carly Hansen, Shih-Chieh Kao

**Affiliations:** Environmental Sciences Division, Oak Ridge National Laboratory, Oak Ridge, TN USA

**Keywords:** Hydrology, Hydroelectricity

## Abstract

First published in 2022, the RectifHyd dataset provides hydrologically consistent estimates of monthly net generation for approximately 1,500 hydropower plants in the United States, addressing a gap in industrial surveys that have collected monthly generation data from only ~10% of plants post-2003. Here we present RectifHydPlus—an extended and enhanced dataset that improves on both the proxy information and temporal downscaling methodology adopted in RectifHyd. In addition to providing updated estimates of historical monthly generation for 590 plants with >10 MW nameplate capacity from 1980 through 2019, RectifHydPlus adds a hydrological control dataset that isolates the influence of historical water availability on generation. The new hydrological control dataset is suited to applications seeking to represent the capabilities of the contemporary fleet subject to historical interannual variability in climate. RectifHydPlus also includes a forty-year, daily-resolution, spill-adjusted water release time series for each dam, allowing users to aggregate generation estimates to the desired temporal resolution.

## Background & Summary

### Need for RectifHydPlus

**Fig. 1:**
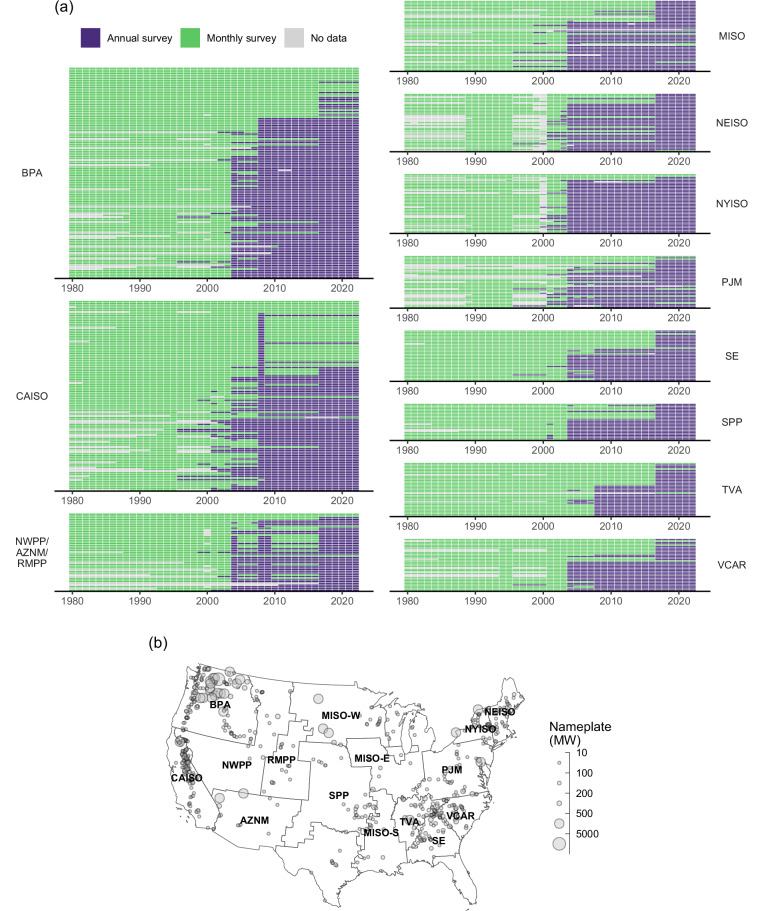
Deterioration of hydropower observational data resolution over time. In panel (**a**) each row represents a hydropower plant, with plants ordered by nameplate (largest at top) within each region. Annual survey refers to collection of data at annual resolution only. Monthly data provided by EIA for these plants/years is statistically imputed, not observed. Panel (**b**) shows balancing regions and plant locations of all 590 plants in this study.

Historical records of monthly electricity generation from hydropower plants inform analyses of hydroclimatic risks to electricity supply^1,2^ and provide essential model inputs for grid reliability and capacity build-out simulation studies^3,4^. In the United States, monthly electricity generation records are collected and distributed each year by the U.S. Energy Information Administration^5^. Since 2003, approximately 90% of U.S. hydropower plants have been surveyed only for annual rather than monthly net generation totals (Fig. 1). To approximate monthly information, the EIA downscales each plant’s annual total energy to monthly energy by inferring the distribution of energy within each year from summed monthly generation across the small sample of surveyed plants within the same administrative region (either the state or, if the number of surveyed plants available within the state is less than five, the census region). Lacking direct representation of local water availability—which is by far the most important driver of seasonal variation in hydropower generation—this approach can yield inaccurate monthly hydropower generation estimates^6^.

The RectifHyd dataset was a first attempt to construct hydrologically consistent monthly hydropower generation estimates for a large sample of U.S. hydropower plants^6^. RectifHyd uses local proxies of generation (i.e., reservoir release data or, if those are unavailable, river discharge at a downstream location) to downscale observed annual generation to monthly. Since its publication, RectifHyd has supported a variety of research applications, including: validation of hydropower simulations in large-scale hydrological models^7^, computation of hydropower energy budgets for production cost models and resource adequacy assessments^8,9^; development of hydropower projections for capacity expansion models^10,11^; computation of metrics for analyzing drought impacts on hydropower generation^12^; calibration of large-scale hydropower model parameters^13^; evaluation of plant-level, monthly hydropower models^14^; and as the basis for further downscaled (weekly) hydropower estimates^15^.

We observe from these recent applications that most analysts using historical hydropower data seek not the record of observation per se, but rather a representation of sub-annual generation from existing plants as a function of varying climate and water availability. Those conducting grid reliability studies seek to represent the behavior of existing infrastructure subject to varying climate conditions, for instance. Historical observations of generation may be inadequate for this purpose as they can be conflated by a range of factors relating to the evolution of plant infrastructure and operations. To illustrate, a plant that adds a unit to double its nameplate capacity will tend to generate much more energy post-upgrade. Conversely, a hydropower plant that increases environmental flows following a relicensing will tend to generate less energy after that determination. Not only have most US plants undergone capacity change in the last forty years, but operational change and infrastructural wear and tear has had a significant impact on generation across the fleet of hydropower plants^16^. An observational record of plant generation is thus prone to misrepresenting the capabilities of the contemporary fleet subject to historical hydroclimatic variation.

Our aims in creating RectifHydPlus are two-fold. First, we aim to supplement the historical monthly hydropower generation estimates in RectifHyd with a new hydrological control dataset that removes the historical influence of capacity and operational change on generation. This new version of the data is designed to support studies requiring representation of the generating variability of plants as they are configured today. Our second aim is to improve the accuracy of the annual to monthly downscaling procedure in RectifHyd. Although RectifHyd offers a significant advance over the procedure adopted by EIA^6^, further improvements to the temporal downscaling are available through collection and synthesis of more representative water data to serve as a proxy for monthly generation. RectifHydPlus achieves this through the addition of new supporting data and models, including an enhanced procedure for estimating spilled energy at each plant (see *Method* for details and Table 1 for comparison with version 1). Validation using monthly observed generation (available widely for the 20^th^ century portion of the record) demonstrates the benefits of these additions, with significant, regionally robust improvements over RectifHyd (see *Technical Validation*).

**Table 1:** RectifHydPlus provides both historical and hydrology control monthly generation estimates for a forty-year period, while improving on downscaling accuracy of RectifHyd with new proxy data and an updated spill procedure (*new proxy introduced in RectifHydPlus).

	RectifHyd	RectifHydPlus
Annual data from which temporal downscaling is performed	Observed annual total net generation.	Annualized time series 1: Observed annual total net generation.Annualized time series 2: Hydrologically controlled annual total net generation.
Period covered	2001–2022 (23 years)	1980–2019 (40 years)
Number of plants covered	~1500	590 (nameplate >10 MW)
Proportion of total CONUS hydropower nameplate capacity represented	99.0%	95.6%
Percentage of downscaled generation based on:		
1. Observed reservoir release records	12%	17%
2. Observed river discharge immediately below dam*	0%	18%
3. Simulated river discharge at dam (run-of-river plants only)*	0%	17%
4. Simulated reservoir release*	0%	22%
5. River discharge at the HUC4 outlet	88%	22%
Spill procedure	Arbitrary Q_90_ cap applied to daily flow	Flow quantile cap calibrated using pre-2001 observations

## Methods

### Legacy downscaling approach

Our approach to downscaling annual hydropower generation data to monthly builds upon the downscaling methodology developed for RectifHyd^6^. This legacy approach may be summarized as follows. For each plant and year, we have an annual total net generation (MWh) which must be allocated among calendar months in a way that respects the principal driver of monthly hydropower—namely, water availability. To do this, RectifHyd employs two different water-related proxies of hydropower generation: daily reservoir release (if available) and daily streamflow at the outlet of the HUC4 basin in which the plant is located (if reservoir release time series are unavailable). In each year, the observed annual net hydropower generation is allocated among months according to how the water proxy is allocated among those months. In other words, if 15% of total water released from a given dam in year Y occurs in January of year Y, then 15% of the annual hydropower for year Y will be allocated to January. There is one important nuance, which is that before monthly total water volumes are constructed from daily flow records for downscaling, a cap is imposed on the daily time series to account for spill. The cap is set at the 90^th^ percentile of the daily flow record. The new RectifHydPlus dataset improves upon this legacy procedure through the application of better proxy data and with the introduction of a more locally relevant spill adjustment procedure for each dam (Table 1).

### Overview of new downscaling approach

**Fig. 2:**
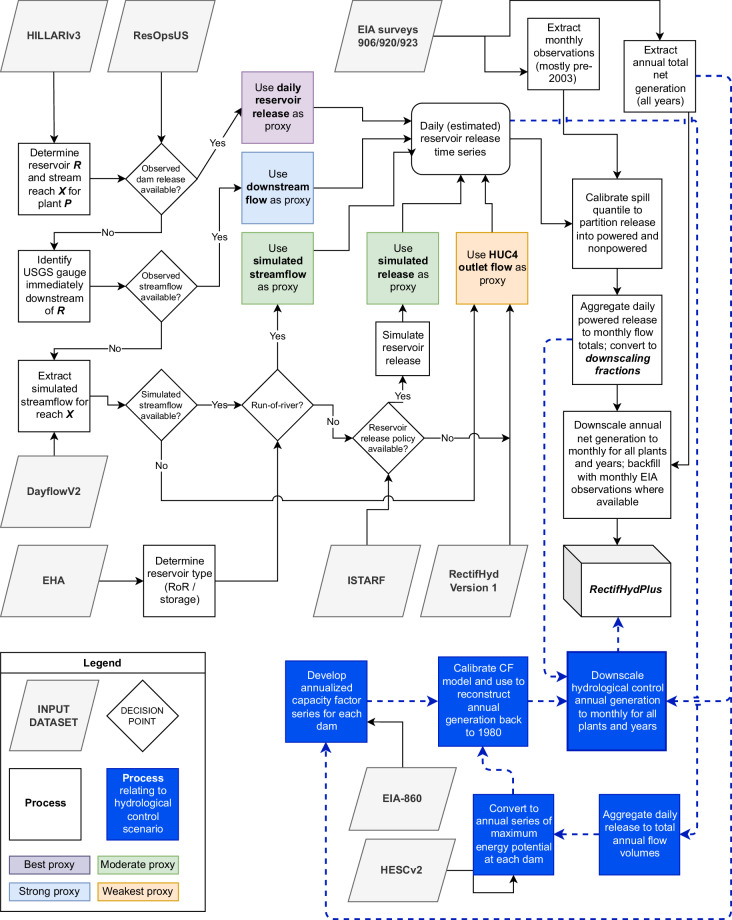
Process flow diagram outlining key inputs, decisions, and processes in the creation of RectifHydPlus. Key input data are Hydropower Infrastructure – Lakes, Reservoirs, and Rivers (HILARRI) Version 3^19^; ResOpsUS^17^; Dayflow Version 2^23^; Existing Hydropower Assets (EHA) Plant Database^22^; Inferred Storage Targets and Release Functions (ISTARF) for CONUS reservoirs^24^; RectifHyd Version 1^6^; EIA surveys 906/920/923^5^; EIA survey 860^18^; Hydropower Energy Storage Capacity Dataset, Version 2^21^.

To prepare RectifHydPlus, we first identify target plants (i.e., CONUS plants with nameplate >10 MW) and then collate reported annual net generation totals from dozens of EIA files (see *Plant selection and annual data preparation*). We create unique plant identifiers (RectifHydPlus ID, or “RHPID”) and map each plant identifier to other identifiers for the dam, reservoir, river reach, and watershed (see *Mapping hydropower plants to dams, reservoirs, and rivers*). This mapping allows us to connect plants to the site characteristics and hydrologic time series data required to generate new proxy information for downscaling annual generation to monthly. For each dam, we attempt to create a daily water release time series from each of five different proxies: observed reservoir release (best proxy), river discharge immediately downstream of the dam (strong proxy, but potentially influenced by conflating inflows between dam and gauge), simulated flow at the dam location applicable at run-of-river plants only (good/moderate proxy), simulated reservoir release (good/moderate proxy), and observed river discharge at the HUC4 outlet (weakest proxy, accounting 22% of downscaling in RectifHydPlus—down from 88% in RectifHyd Version 1)—see *Development of daily water release*. We select the best available proxy for each dam and then adjust the daily time series to account for spill (i.e., water releases that bypass the turbines) before aggregating to a monthly timescale for use in downscaling. This is similar to the RectifHyd approach with the key difference that here we adjust the spill percentile separately for each dam (*Spill adjustment and aggregation to monthly powered release totals*). With the spill-adjusted monthly proxy data prepared, downscaling is performed on both the observed annual generation totals and a synthesized set of annual generation totals for the hydrological control scenario (see Hydrological control scenario), creating two separate datasets of monthly generation: ***RectifHydPlus_HIST*** (historical) and ***RectifHydPlus_CTRL*** (hydrological control) (Fig. 2). Code required to reproduce RectifHydPlus are organized in a formal data pipeline and shared in an open repository (see *Code Availability*).

### Plant selection and annual data preparation

The hydrologic data used to develop proxies of generation in RectifHydPlus are currently unavailable for Alaska and Hawaii. We therefore select RectifHydPlus plants by first filtering all plants in the most recent EIA-923 dataset^5^ for conventional hydropower plants located in the contiguous United States. We then remove plants with nameplate capacity less than 10 MW—leaving 607 unique EIA identifiers. Removal of small plants (<10 MW) provides stronger confidence accuracy of the downscaled monthly data. This is because the proxy data required to downscale annual generation to monthly is often unavailable or too unreliable for smaller streams. The new proxies introduced in RectifHydPlus require accurate linkage of plant to river reach—an increasingly challenging task with smaller plants, which are often located on conduits or canals rather than the natural river network. Almost no small plants (<10 MW) in the legacy RectifHyd dataset benefit from strong data proxy (i.e., reservoir release from ResOpsUS^17^), and the alternative proxy (observed river discharge at the HUC4 outlet) often misrepresents hydrologic conditions of small tributaries on which smaller plants are often located. This 10 MW capacity cutoff means RectifHydPlus features considerably fewer plants than the legacy dataset (RectifHyd Version 1 features 1,492 plants). Nonetheless RectifHydPlus represents 96% of US installed hydropower capacity; omission of small plants <10 MW should therefore not degrade the value RectifHydPlus for most practical applications. Applications involving regional analysis of hydropower variability, or development of input data for power system models, are better served by improved accuracy at the largest facilities than by inclusion of all possible plants.

Next, the 607 EIA identifiers are used to extract historical plant capacities from EIA-860 (all years 1980 through 2022)^18^ and annual net generation totals from forms EIA-759 (covering years 1980–2000), EIA-906 (1989–2000 nonutility), EIA-920 (2001–2007), and EIA-923 (2008–2022)^5^. Some plants in the EIA datasets are split across two or more EIA identifiers. For instance, Hoover Dam has separate EIA identifiers for the Arizona (EIA ID 154) and Nevada (EIA ID 8902) powerhouses located on either bank of the Colorado River. While state-level reporting requirements and political reasons can motivate splitting of plant data in this way, the appropriate representation of a plant for the purposes of RectifHydPlus is one plant identifier per dam/reservoir. We therefore combine Hoover Dam (and other such cases) to a single plant, creating a new identifier (the RectifHydPlus identifier, or “RHPID”) that combines the plant’s EIA IDs and plant names (inherited from HILARRI Version 3) to a single string (e.g., Hoover Dam becomes “154/8902_HOOVER DAM (NV & AZ)”). This leaves 590 unique RHPIDs to be mapped to hydrologic data and site characteristics.

### Mapping hydropower plants to dams, reservoirs, and rivers

We link each RHPID to its respective dam, reservoir, and river reach using HILARRI version 3^19^. HILARRI maps US power plants to identifiers in other supporting datasets—namely the Global Reservoir and Dams (GRaND) ID (providing a link to reservoir data and specifications at 328 dams^20^), National Inventory of Dams (NID) ID (linking to dam specifications at 526 dams), Hydropower Energy Storage Capacity (HESC) dataset (linking to estimates of hydraulic head at some dams^21^), and the Existing Hydropower Assets (EHA) database^22^ (linking to plant locations and modes of operation, including identification of run-of-river facilities). HILARRI also provides the USGS station ID for flow gauges immediately downstream of the dams, providing a valuable indicator of reservoir release that serves as a proxy for generation. This mapping effort culminates in a plant to water data mapping table (one row per 590 RHPIDs) with the following columns: RHPID, GRaND_ID, NIDID, COMID, HUC12, HYD_HEAD_m, OPERATING_MODE, LON, LAT, USGS_GAUGE. The plant to water data mapping table is included in the RectifHydPlus data release.

### Development of daily water release data

Downscaling of annual hydropower to monthly resolution in RectifHydPlus assumes that the distribution of monthly energy generation totals throughout the year can be accurately represented by the distribution of monthly powered water release volumes at the dam (i.e., total release minus spill). A potential limitation with this approach is that powered water releases neglect possible variation in the rate of conversion of flow to power. Such variation is brought about by varying unit efficiencies or by fluctuations in hydraulic head driven by changing headwater and tailwater elevations at the dam. Lacking turbine efficiency curves and pool elevation time series for headwater and tailwater, such details cannot be readily incorporated into RectifHydPlus. Results achieved for RectifHyd Version 1 demonstrate that monthly powered release volumes (where available) are an excellent proxy for monthly generation. A primary aim of RectifHydPlus is thus to expand the number of plants with accurate representation of this variable.

In RectifHydPlus, we use the closest available representation of water release for each plant and year of operation. This means some plants could be associated with multiple sources of water release data over the 40-year period. There is no requirement for a consistent data source at a given plant, since each year of operation is downscaled independently. As with the legacy version of RectifHyd, direct observations of reservoir release are the most desirable data. We adopt daily reservoir water records from ResOpsUS^17^, which combines US reservoir operations records obtained by web-scraping from various agency data portals (CDEC, USACE, USBR, TWDB) with data collected by national-scale survey of dam operators. If, for any given year of operation at a plant, the observed reservoir release data are unavailable, RectifHydPlus adopts the next best available estimate of reservoir release: river discharge recorded immediately downstream of the dam. If these are missing, the next closest representation of water release is selected, and so on. The priority order for water release data is: (1) direct observation of release from ResOpsUS, as described above; (2) River discharge at USGS gauge within 10 km downstream of the dam (expected to be near identical to a direct release observation unless the discharge is interrupted between dam and gauge, such as by tributary inflow or significant discharge or withdrawal); (3) simulated river discharge at the dam location, extracted from Dayflow Version 2^23^, and used only if the plant is type run-of-river; (4) simulated reservoir release from the dam, based on water release policies defined in the Inferred Storage Targets and Release Inference Functions (ISTARF)^24^; and (5) discharge from the USGS gauge at the HUC4 outlet, as adopted in RectifHyd Version 1.

### Spill adjustment and aggregation to monthly powered release totals

Before being used to downscale annual generation, the daily release time series must be adjusted to account for the portion of water that bypasses the turbine. This is known as spill. A common approach to spill estimation in a large-scale study is to cap the daily flow at an arbitrary percentile intended to represent turbine flow capacity. This is the approach applied in the RectifHyd legacy dataset, which assumes a cut-off of the 90^th^ percentile—meaning flow is constrained by a maximum value of the 90^th^ percentile of daily flow. In RectifHydPlus we improve on this adjustment by calibrating the percentile at which total release is capped to adjust for spill. Our calibration procedure is as follows. For a given percentile, one can compute daily powered release volumes (i.e., release with spill removed), sum these to give monthly powered release volumes, assign to each month a fraction based on how much each month’s volume contributes to the total annual powered release volume, then apply those same fractions to the annual generation to give monthly generation. Since monthly generation observations are widely available in EIA survey data prior to 2003, one can optimize the spill percentile value to give the best representation of monthly generation in those years. The optimal spill fraction can then be applied across all years at the dam. In RectifHydPlus, this spill quantile is found by Brent’s method^25^, minimizing the root mean squared error between observed and modeled monthly generation. With the spill quantile calibrated for a plant, the final daily powered release time series is computed and then summed to monthly water volumes to be used directly in downscaling annual generation to monthly in all years.

**Fig. 3:**
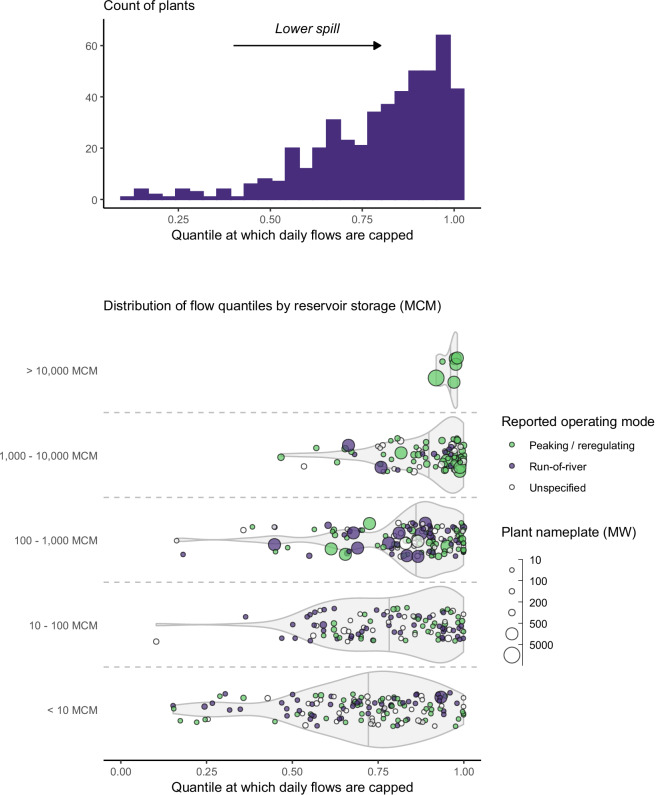
Calibrated spill quantiles for each plant represented in RectifHydPlus. Upper panel gives the overall distribution. Lower panel provides the same data, splitting by reservoir storage category (larger storage is associated with less spill).

The above procedure reveals a median spill percentile of 0.85 (85^th^ percentile) across all plants (Fig. 3). Approximately 37% of plants are represented with a spill quantile larger than 0.9 (meaning spill occurs on fewer than 10% of days). Reservoirs with large storage tend to be associated with infrequent spilling (i.e., higher spill percentile). Smaller reservoirs often have insufficient capacity to capture high inflow events, while small plants (by nameplate) will have limited ability to convey high flows through turbines. High spill frequency thus tends to be more common in small storage dams with low plant nameplate. Analysis of monthly generation estimates derived using the new calibrated spill values demonstrates the value of the approach, with significance improvements in generation accuracy relative to the 90^th^ percentile assumption of the legacy dataset (see Technical Validation).

### Hydrological control scenario

RectifHydPlus includes a new hydrological control scenario (“RectifHydPlus_CTRL”) to complement to the historical generation estimates (“RectifHydPlus_HIST”). These new data are derived by parameterizing for each plant an annual flow to capacity factor model, which is then used to simulate annual capacity factor as a function of hydrological conditions from 1980 till present day. Modeled annual capacity factors are converted to generation assuming contemporary nameplate capacity and then downscaled to monthly generation using the monthly fractions derived for historical generation downscaling described above. The annual capacity factor model is the Energy Limited Capacity Factor Curve (ELCFC) introduced in^16^:

$${{CF}}_{{yr}}=\frac{{E}_{{yr}}}{\widetilde{E}}=1+\,{\varphi }_{{yr}}-{(\gamma +{{\varphi }_{{yr}}}^{\tau })}^{1/\tau }$$

$$\varphi =\frac{{\hat{E}}_{{yr}}}{\widetilde{E}}=\frac{\rho \times g\times h\times {Q}_{{yr}}\times (2.78\times {10}^{-10})}{\widetilde{E}}$$

The model relates a plant’s capacity factor (annual energy ${E}_{{yr}}$ divided by annual maximum energy $\widetilde{E}$—both in units of MWh) to its full capacity potential (annual potential maximum energy ${\hat{E}}_{{yr}}$ implied by available water, divided by annual maximum energy $\widetilde{E}$) using two calibrated parameters, $\gamma$ and $\tau$, where $\rho$ is the density of water (1000 kg/m^3^), $g$ is gravitational acceleration (9.81 m/s^2^), $h$ is the hydraulic head of the plant (m), and ${Q}_{{yr}}$ is the annual total inflow volume (m^3^). The constant $2.78\times {10}^{-10}$ converts annual potential maximum energy from Joules to MWh. Model reasoning, visualization, and validation are provided in^16^. Here we note that this model has been demonstrated to be superior to linear models of annual hydropower while obeying important physical constraints relating to the capacity limit of the plant (the model will never return CF > 1) and the energy potential in flow (the model cannot produce energy that exceeds the energy potential given annual flow).

Data are prepared for model calibration as follows. For each RHPID, historical plant capacities (MW) are converted to maximum annual generation $\widetilde{E}$ (MWh) by multiplying by number of hours each year (accounting for leap year differences). Actual annual generation divided by maximum annual generation then gives the time series of annual capacity factors. Annual water availability (${Q}_{{yr}}$) is then determined using summed daily flow volumes from either ResOpsUS^17^—if available for the full period 1980–2019—or Dayflow Version 2^23^. Hydraulic head, $h$, is from the Hydropower Energy Storage Capacity (HESC Version 2) dataset^21^. Model calibration is performed for the period 2005 through 2019 (i.e., the latter 15 years of the 40-year time series), capturing capacity factors relevant to contemporary operations. Parameters are fitted using the Limited-memory Broyden-Fletcher-Goldfarb-Shanno algorithm, known as “L-BFGS”^26^. The fitted model is then used to simulate annual capacity factor for the full period 1980 through 2019. Finally, we multiply simulated capacity factors by contemporary (year 2022) plant nameplate to give the annualized hydrological control hydropower generation time series.

**Fig. 4:**
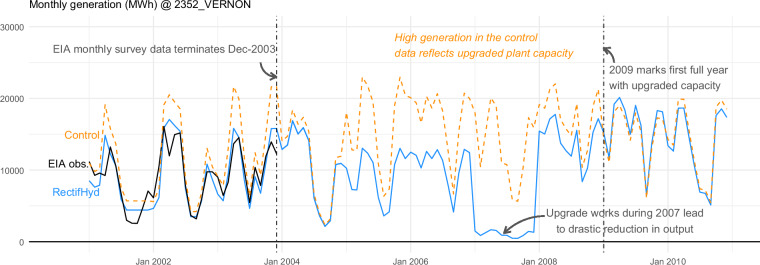
RectifHydPlus provides historical and hydrological control scenario monthly generation time series. This example shows a slice of the data for Vernon hydropower plant (EIA 2352). The data illustrate RectifHydPlus model performance (during pre-2004 period for which actual monthly observations are available) and show significant differences between historical estimates and hydrological control scenario, with the former reflecting reduced capacity as well as a major outage in 2007.

To convert to monthly generation, the hydrological control data are assigned with the same downscaling factors derived for the historical data in RectifHydPlus, giving a complete set of monthly generation totals for all 590 plants. For illustrative purposes, a comparison of RectifHydPlus historical generation (RectifHydPlus_HIST) versus RectifHydPlus hydrological control generation (RectifHydPlus_CTRL) is given in Fig. 4, depicting results for the Vernon hydropower plant on the Connecticut River (EIA identifier 2352). This plant underwent a significant capacity upgrade in 2007/2008, with nameplate increasing by 71% from 20.4 MW to 34.9 MW. As a result, we see significantly larger generation in RectifHydPlus post-2009, which is reflected in the hydrological control case back through the earlier years of record. This example also shows how the hydrological control case omits historical periods of outage, such as in 2007 when output was cut during the upgrade works.

**Fig. 5:**
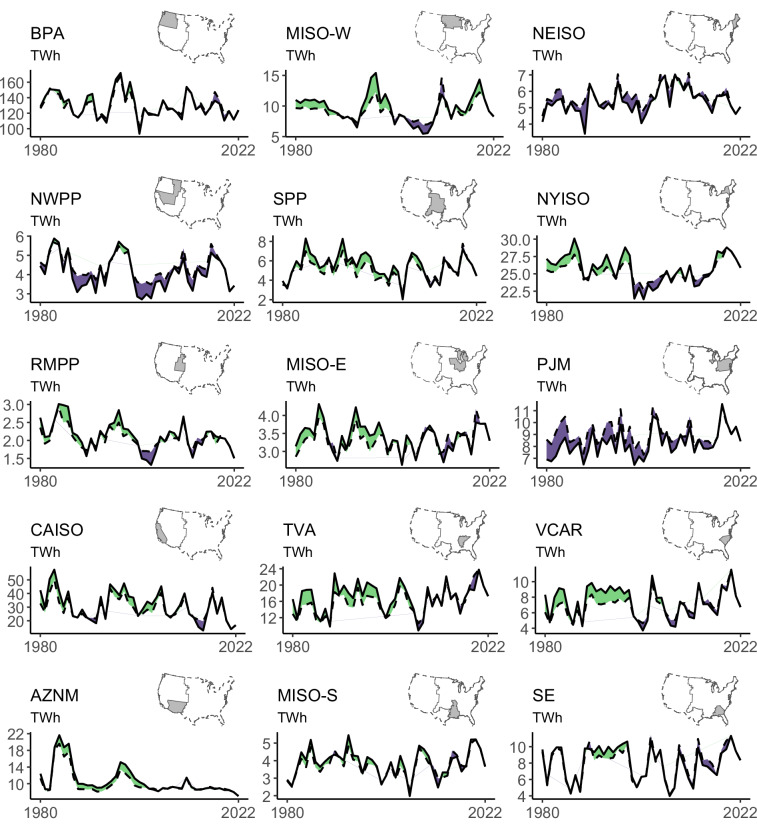
Annualized hydropower totals for fifteen balancing authority regions (based on the Regional Energy Deployment System, ReEDS, model regions). Solid line is actual historical annual generation while dashed line represents the hydrological control. Light green indicates that the hydrological control scenario generation is lower than actual historical, while dark blue indicates that hydrological control scenario generation exceeds actual historical generation.

The RectifHydPlus_CTRL generation estimates can also be smaller than RectifHydPlus_HIST. If a plant has experienced a capacity downgrade (e.g., a unit retirement) or if its efficiency has declined significantly, the hydrological control case will produce lower generation relative to historical in the earlier portions of the record. At the aggregated level of power balancing authorities, the data show a general tendency for the annual hydrological control generation to be marginally lower than annual historical generation (Fig. 5). This mainly reflects the generally reduced power generating capabilities of plants despite capacity upgrades, attributed in^16^ to plant wear and tear and shifting operating policies in support of nonpower objectives. Exceptions are the NWPP, PJM, and NEISO regions, for which the hydrological control case produces marginally increased annual generation relative to historical.

## Data Records

RectifHydPlus^27^ is openly available at: https://hydrosource.ornl.gov/dataset/rectifhydplus.

RectifHydPlus includes three tables of monthly hydropower net generation totals. All three tables have 23,600 data rows (590 hydropower dams multiplied by 40 years), featuring the following columns: *RHPID* (the RectifHydPlus identifier, which combines the numeric EIA ID and plant name), the year (1980–2019), a column for each calendar month (Jan – Dec) consisting of energy generation estimates for the respective months (in MWh), and a label indicating the proxy data used to downscale the annual estimate to monthly, along with the quality of that proxy (Best, Strong, Good/Moderate, Weak). The three tables are named: “RectifHydPlus_HIST-***Backfilled***_MWh.csv”, “RectifHydPlus_HIST-***Validation***_MWh.csv”, and “RectifHydPlus_***CONTROL***_MWh.csv”. The “backfilled” data provide monthly generation totals downscaled from annual generation observations, with available observed monthly EIA observations backfilled, replacing RectifHydPlus estimated generation primarily in the period 1980–2002 (after which most plants became surveyed at annual resolution only). For the backfilled rows, the data quality label is replaced with “X. EIA monthly survey (observation),” allowing users to easily identify which data are observed versus downscaled. The “validation” data do not include this backfill and can thus be used alongside the “backfilled” data to validate the RectifHydPlus downscaling procedure (by comparing downscaled values to corresponding values in the backfilled dataset). Finally, the “control” table contains monthly generation for the hydrological control case.

In addition to the three main data tables, RectifHydPlus includes the following supplementary data files:

- A reference table that links each RectifHydPlus identifier (“RHPID”) to relevant water-related datasets,
- The daily, spill-adjusted powered release time series created for each of the 590 dams,
- A compressed archive containing all code required to reproduce RectifHydPlus (all three tables), organized as a data pipeline using the R {targets} framework, and
- A compressed archive containing all input data used to create RectifHydPlus, organized for seamless entry into the data pipeline.

## Technical Validation

**Fig. 6:**
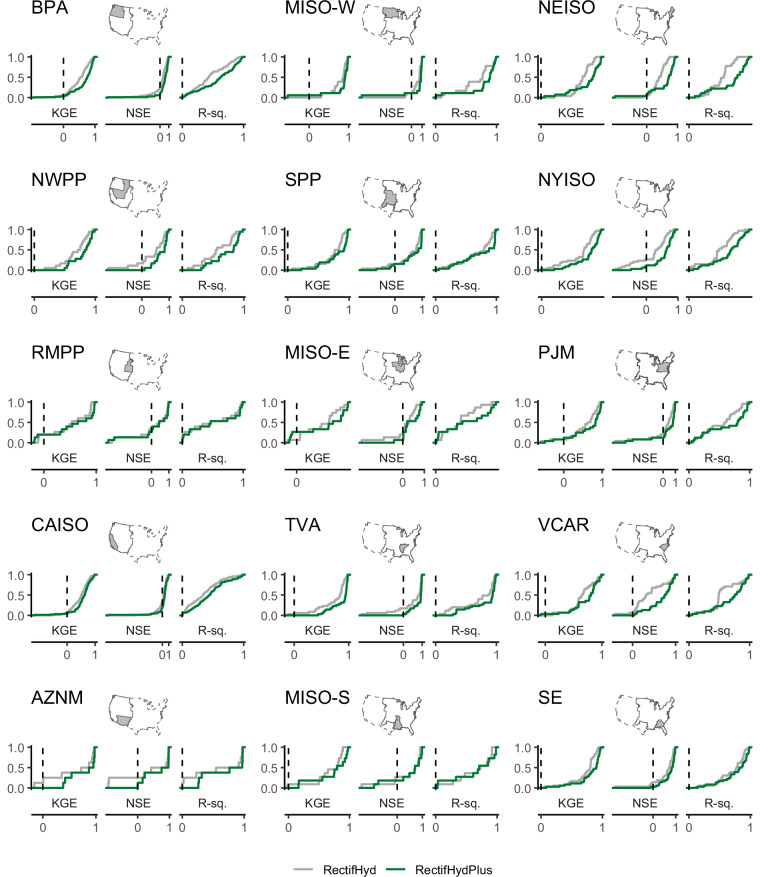
Performance of RectifHydPlus relative to RectifHyd across three performance metrics. Results are shown as cumulative distribution functions for each balancing region. Distribution of performance within each region reflects performances across plants in the 510-plant validation set during the 21-year period 1980–2000.

We evaluate the performance of RectifHydPlus using monthly observations available for 510 plants over the 21-year period 1980–2000. In our evaluation, we first confirm that there is zero bias in the data by ensuring that the annual mean of monthly generation for each year and each plant in RectifHydPlus is equal to that of the observation. This simple check confirms that annual observed generation totals are preserved in RectifHydPlus. Each year of downscaled data associated with each plant is evaluated using the coefficient of determination (R^2^ value), the Nash-Sutcliffe Efficiency (NSE), and the Kling-Gupta Efficiency (KGE). We also compute these performance values (same set of plants and years) in RectifHyd Version 1, allowing for analysis of the improvements offered in RectfHydPlus (Fig. 6). Since Proxies A and E are both present in RectifHyd Version 1, performance changes across plant-years in these categories can be attributed to the updated spill procedure in RectifHydPlus. Since proxies B, C, and D are absent from RectifHyd Version 1, performance changes across plant-years in these categories can be attributed to both the updated spill procedure and the availability of better proxy information.

The evaluation shows that RectifHydPlus offers significant and regionally robust performance improvements over RectifHyd Version 1 (Fig. 6). Across all 510 plants (with results reported here along with 10^th^ and 90^th^ percentiles of each score in parentheses), the median NSE score for RectifHydPlus is 0.67 [0, 0.96]—a significant increase from RectifHyd version 1 with median NSE score of 0.416 [−0.56, 0.90] across the same plants. Similar improvements are found for KGE—with 0.78 [0.30, 0.96] for RectifHydPlus against 0.65 [0.16, 0.89] for RectifHyd—and for the coefficient of determination, with 0.69 [0.97, 0.16] against 0.50 [0.91, 0.09]. The proportion of plants with improved performance in RectifHydPlus is 80%, 76%, and 78% for NSE, KGE, and R-squared, respectively.

Most improvements are attributable to the three new proxies introduced in RectifHydPlus (Table 2). Median R^2^ values increase from 0.31 to 0.83, 0.44 to 0.69, and 0.43 to 0.69, across plant-years with the addition of Proxies B, C, and D, respectively. Similarly, median NSE values increase from 0 to 0.78, 0.02 to 0.59, and 0.19 to 0.57, respectively. The jump in performance reflects the proxy data used in RectifHydPlus being more likely to represent water releases than the gauge data employed in RectifHyd Version 1. For the evaluations concerning proxy A (ResOpsUS water releases) and Proxy E (flow gauge at the HUC4 outlet), versions 1 and 2 are relying on the same proxy information. For these plants, the marginal improvements in downscaling performance with RectifHydPlus are attributable to the new spill procedure.

**Table 2:** Improved downscaling in RectifHydPlus attributable to new proxy data and bespoke spill procedure.

	RectifHyd median score	RectifHydPlus median score	% of plant-years improved with RectifHydPlus
**Proxy A** (2,405 dam-years)			
NSE	0.89	0.96	77%
KGE	0.81	0.92	76%
R squared	0.92	0.97	72%
Proxy B* (1,698 dam-years)			
NSE	−0.01	0.78	86%
KGE	0.23	0.75	76%
R squared	0.31	0.84	80%
Proxy C* (1,169 dam-years)			
NSE	0.02	0.60	84%
KGE	0.42	0.70	78%
R squared	0.44	0.69	76%
Proxy D* (2,446 dam-years)			
NSE	0.19	0.57	73%
KGE	0.44	0.67	70%
R squared	0.43	0.69	69%
**Proxy E** (1,851 dam-years)			
NSE	−0.03	0.19	73%
KGE	0.27	0.41	62%
R squared	0.33	0.37	60%

Performance scores computed for 510 plants and 21 years (10,700 plant-years). Performance scores are computed using observed monthly data available 1980 – 2000. *Data are split according to the proxy data used to downscale annual generation in RectifHydPlus (not RectifHyd, which lacks proxies B, C, and D)—meaning results reported for RectifHyd in proxies B, C, and D reflect performance in the absence of those new proxies.

RectifHydPlus also improves the distribution of performance values across all balancing regions (Fig. 6). Regions with the largest overall gains are the Pacific Northwest (BPA), west and east Midcontinent (MISO-W and MISO-E), New England (NEISO) and New York (NYISO). Scores throughout the California ISO are among the lowest, reflecting the difficulty of generating accurate water availability estimates for this region. Specifically, a disproportionately large fraction of dams in California are off-stream, or they rely on non-natural inflows from canals and other water conduits.

**Fig. 7:**
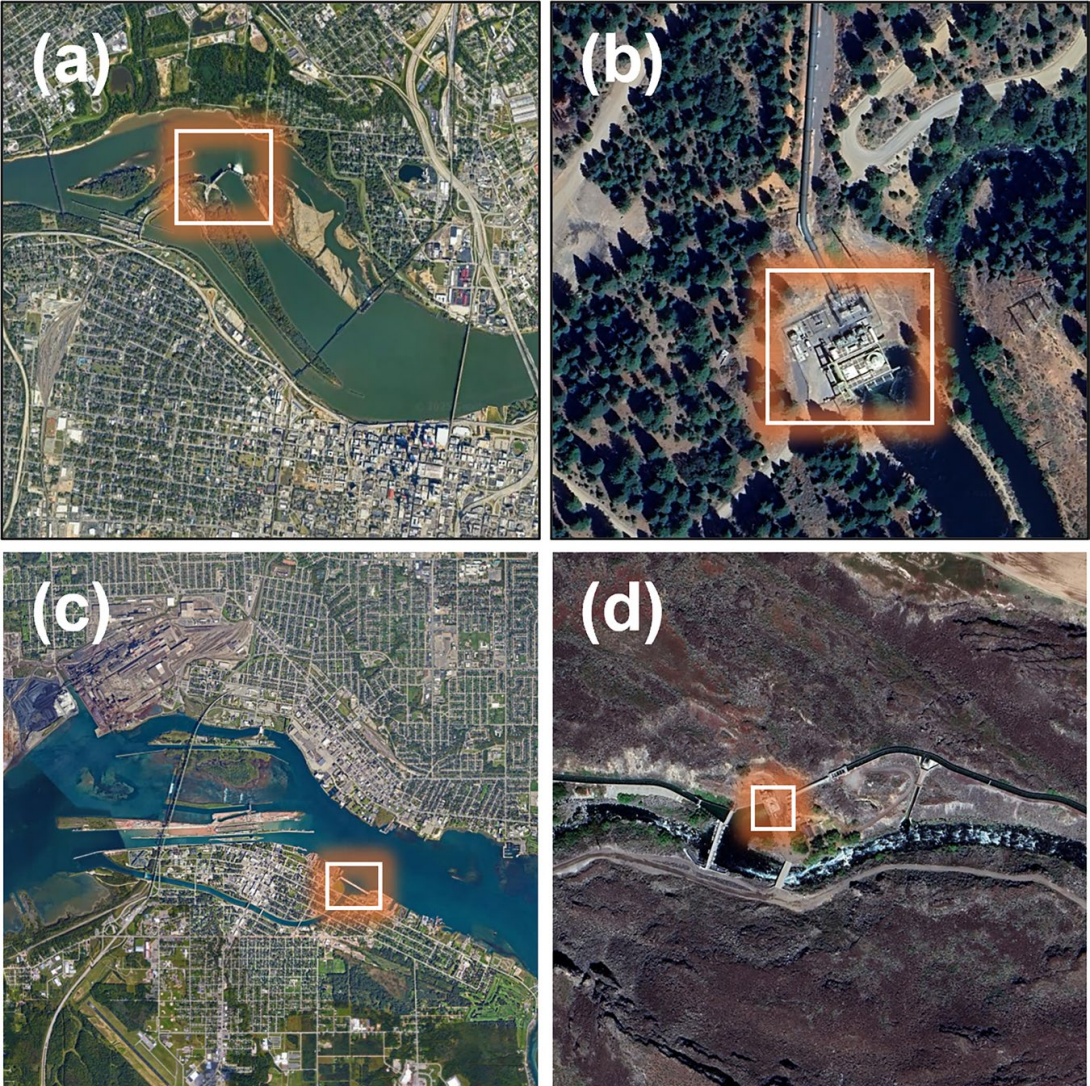
Atypical infrastructural settings. Examples of plants in RectifHydPlus with weak performance owing to unusual features of the infrastructure: (**a**) “1365_OHIO FALLS”, (**b**) “221_BUTT VALLEY”, (**c**) “1751/1865_EDISON SAULT & ST MARYS FALLS”, and (**d**) “815_LOWER MALAD”.

There are 53 test plants exhibiting negative NSE scores in RectifHydPlus, which is about half the equivalent number for RectifHyd. While this represents a significant improvement in the new data, residual negative NSE values highlight some inadequacies in the downscaling which should be understood and explained. Unsurprisingly, most of the weak performances in RectifHydPlus are associated with a high-quality proxy being unavailable. However, there are some cases that perform poorly in validation despite being linked to strong proxy data. Such cases are characterized by atypical infrastructural settings, including run-of-river plants located on split channels (e.g., Ohio Falls, Kentucky, shown in Fig. 7a), off-stream plants fed by conduits (common in California and shown for Butt Valley in Fig. 7b), and plants generating flows from different types of controlled diversions off the main stem of the river (e.g., Saint Mary’s Falls, Michigan, shown in Fig. 7c; Lower Malad Dam, Idaho, shown in Fig. 7d). These edge cases provide an impetus for further data collection and improvements to RectifHydPlus downscaling methodology in future releases.

## Data Availability

RectifHydPlus can be reproduced in its entirety from a single, comprehensive data pipeline coded in the R {targets} framework. The reproducible data pipeline is held at the following GitLab repository https://code.ornl.gov/turnersw/rectifhydplus. Inputs to the data pipeline are available as a compressed archive in the RectifHydPlus data repository.
